# Identification and Characterization of P0 Protein as a Vaccine Candidate Against *Babesia divergens*, Blood Parasite of Veterinary and Zoonotic Importance

**DOI:** 10.3389/fvets.2021.795906

**Published:** 2022-01-07

**Authors:** Shimaa Abd El-Salam El-Sayed, Mohamed Abdo Rizk, Haitham Eldoumani, Shimaa Sobhy Sorour, Mohamad Alaa Terkawi, Mahmoud AbouLaila, Ikuo Igarashi, Mohamed Z. Sayed-Ahmed

**Affiliations:** ^1^National Research Center for Protozoan Diseases, Obihiro University of Agriculture and Veterinary Medicine, Obihiro, Japan; ^2^Department of Biochemistry and Chemistry of Nutrition, Faculty of Veterinary Medicine, Mansoura University, Mansoura, Egypt; ^3^Department of Internal Medicine and Infectious Diseases, Faculty of Veterinary Medicine, Mansoura University, Mansoura, Egypt; ^4^Department of Anatomy, Faculty of Veterinary Medicine, Mansoura University, Mansoura, Egypt; ^5^Department of Parasitology, Faculty of Veterinary Medicine, Kafrelsheikh University, Kafr El-Shaikh, Egypt; ^6^Department of Parasitology, Faculty of Veterinary Medicine, Damanhour University, Damanhour, Egypt; ^7^Department of Clinical Pharmacy, College of Pharmacy, Jazan University, Jizan, Saudi Arabia

**Keywords:** *Babesia divergens*, P0 protein, vaccine, zoonotic infection, diagnosis

## Abstract

The molecular identification and antigenic characterization of P0 protein in *Babesia divergens*, a blood parasite of veterinary and zoonotic importance, were carried out in this study for use in developing subunit vaccines against *B. divergens* infection. Recombinant protein encoding P0 (BdP0) was developed in *Escherichia coli*, and its antiserum was generated in mice for further molecular characterization. Anti-rBdP0 serum had a specific interaction with the corresponding legitimate *B. divergens* protein, as confirmed by Western blotting and indirect fluorescent antibody tests. ELISA was used to assess the immunogenicity of BdP0 in a group of 68 bovine field samples, and significant immunological reactivity was found in 19 and 20 positive samples of rBdp0 and *B. divergens* lysate, respectively. The *in vitro* growth of *B. divergens* cultures treated with anti-rBdP0 serum was significantly inhibited (*p* < 0.05). Furthermore, after 6 h of incubation with 2 mg/ml anti-rBdP0 serum, the ability of pre-incubated free merozoites to invade bovine erythrocytes was reduced by 59.88%. The obtained data suggest the possible use of rBdP0 as diagnostic antigen and may serve as a vaccine candidate against babesiosis caused by *B. divergens* either in animal or human.

## Introduction

Despite numerous efforts, babesiosis is still one of the most infectious protozoan diseases worldwide. *Babesia* protozoa are apicomplexan eukaryotic tick-transmitted organisms that infect a wide range of hosts, presenting a serious health and economic concern for the cattle industry with a wide range of clinical presentations, from self-healing infections to potentially deadly infections (1, 2). Babesiosis has long been known as an economically significant illness in cattle, but it was only in the last 30 years that several *Babesia* species were recognized as serious pathogens in humans, with *B. divergens* being one of them (3). *B. divergens*, a natural pathogen of cattle, is the main pathogen of human babesiosis in Europe (4).

In the past decades, important progress in discovering a promising antigen to control babesiosis infection has been developed, one of the promising antigens are ribosomal P-proteins, which consist of three main proteins named p0, p1, and p2 (5–7). The P0 protein is found in the cell as part of the ribosomal particle, where it forms a lateral ribosomal element known as the P-stalk structure (5). The activation of translational GTPases during each step of protein synthesis is thought to be the major function of this ribosomal structure (7). P0 (but not P1 and P2) is of vital importance to cells, as demonstrated in *Saccharomyces cerevisiae* (6). P0 has three domains: an N-terminal one that binds to the GTPase-associated region (GAR) of 25S rRNA (8), a central one with at least two distinct areas necessary to bind the P1–P2 and P1–P2 dimers (9, 10), and a highly conserved C-terminal peptide, which is required for the protein activity in translation (11). Moreover, additional so-called extra-ribosomal function was ascribed for the P-protein, showing that this ribosomal proteins can be associated with numerous metabolic processes non-related to the ribosome activity, such as tumorigenesis (12, 13), apoptosis (14) autophagy (15), and pathogenesis of autoimmunological diseases (16). Although a ribosomal component, this protein has been located on the surface of many eukaryotic cells, including many protozoan parasites (17). Because of the surface localization and immunogenicity of P0 proteins, it has been suggested that they may be possible vaccine candidates against *Plasmodium yoellii, Leishmania major*, and *Babesia microti* (18–20). Moreover, *in vitro* studies have demonstrated that anti-P0 antiserum can neutralize *Toxoplasma gondii, Neospora caninum*, and *Babesia bovis* parasites by either inhibiting their growth or blocking cell invasion (20, 21), in addition to their role in the development of immunity against the malaria pathogen. The P0 protein was found on the *Plasmodium* sp. cell wall (17), whereas the P2 protein was localized on the surface of infected red blood cells at an early stage of the parasite development (17, 22). Despite of their important role, the molecular characterization of P0 protein in *B. divergens*, a blood parasite of veterinary and zoonotic importance, has not been done yet. In light of this, the present study investigated the molecular identification and antigenic characterization of the *B. divergens* ribosomal P0 protein in order to produce subunit vaccines to protect against *B. divergens* infection.

## Materials and Methods

### Parasite Strain and Cultivation

A microaerophilic, stationary-phase culture system was used for cultivation of *B. divergens* (German strain) (23) in bovine red blood cells (RBCs) reared in Roswell Park Memorial Institute (RPMI) 1640 medium (Sigma-Aldrich) according to Rizk et al. (23, 24). RPMI 1640 medium was supplemented with 40% normal bovine serum, penicillin G, streptomycin, and amphotericin B (60 U/ml, 60 μg/ml, and 0.15 μg/ml), respectively (all three drugs from Sigma-Aldrich). The parasite was cultivated in 24-well plates at 37°C in an atmosphere of 5% CO_2_ and O_2_ and a 90% N_2_ gas mix. At peak parasitemia, all parasitized red blood cells (pRBCs) were harvested and then kept at −80°C for further use.

### Cloning, Expression, and Production of Mice Antiserum Against rBdP0 and Its IgG Purification

Two oligonucleotide primers, F (5′ GCGAATTCTTGAGAAGTTGTATGACAG-3′) and R (5′-GCCCTCGAGACTTCTCAAGTTTGAGACCG-3′), were used to amplify BdP0 genes (GenBank accession number LC056926.1) from the cDNA by PCR. The resulted amplified gene was digested with Ecor1 and Xho1 enzymes followed by subcloning into a pGEX-4T vector (Amersham Pharmacia Biotech, Madison, CA, USA) and expressed as a glutathione S-transferase (GST) fusion in an *E. coli* DH-5α strain (Amersham Pharmacia Biotech) (an optimal strain for plasmid propagation and stable amplification of the pDNA using plasmid-derived vectors with increasing the insert stability and improving the quality of plasmid DNA) (25). Following normal techniques (26), the recombinant protein was refined using A Glutathione-Sepharose 4B (agarose bead) (Amersham Pharmacia Biotech) and used to produce antisera against rBdP0 and control GST protein in 6-week-old BALB/c mice (*n* = 5). The mice were injected i.p. with 100 μg purified rBdP0 emulsified with complete Freund's adjuvant (1:1), followed by three booster doses of the same antigen emulsified with Freund's incomplete adjuvant (1:1) given at the same route at 14-day intervals. The indirect fluorescent antibody test (IFAT) and Western blotting were used to identify the specific antibodies production in mice sera collected 2 weeks after the last booster. Following that, total IgG was extracted from the mouse serum using a protein A chromatography column, as directed by the manufacturer (Bio-Rad Laboratories, Hercules, CA, USA).

### SDS-PAGE and Western Blotting

The produced recombinant protein was verified using sodium dodecyl sulfate–polyacrylamide gel electrophoresis (SDS-PAGE) with subsequent Coomassie Blue staining R250, while protein antigenicity was confirmed using Western blot analysis as previously described (26, 27).

### Enzyme-Linked Immunosorbent Assay

An enzyme-linked immunosorbent assay (ELISA) was performed on a set of 68 field bovine sera, which were stocked at our laboratory with rBdp0 and *B. divergens* lysate (26–28). The microtiter plates (Nunc, Denmark) were coated overnight at 4°C with 2 μg/ml of the rBdP0 or *B. divergens* lysate using a coating buffer (0.05 M carbonate buffer, pH 9.6). The plates were then blocked with a 3% (w/v) skim milk solution in phosphate-buffered saline (PBS) for 1 h at 37°C. After washing, the plates were incubated with serum samples at a dilution of 1:100 for 1 h at 37°C. The bound antibody was detected by treatment with horseradish peroxidase (HRP)-conjugated (BETHYL, Laboratories, Inc.) anti-bovine IgG (1:4,000) and ABTS [2,2′-azinobis (3-ethylbenzthiazolinesulfonic acid)] (Sigma). The color was allowed to develop at room temperature. The optical density (OD) was measured using the MTP-500 microplate reader (Corona Electric, Tokyo, Japan) at 415 nm. To get the final results, the mean OD value of two readings with the rBdp0 protein was deducted from the mean OD value of two readings with the GST protein. The cutoff values were calculated using 25 non-infected bovine sera.

### Confocal Laser Microscopic Investigation

*B. divergens*-pRBCs were fixed for 30 min in a solution of 95% methanol and 5% acetone (v:v) at 20°C. The Bdp0 cellular localization was then determined using an immunofluorescence standard procedure (26).

### *In vitro* Growth Inhibition Assay

Fluorescence assay was used to perform a growth inhibition assay using *B. divergens* culture reared in RPMI 1640 medium with same culture condition as mentioned above. RBCs infected with *B. divergens* parasite were cultivated for 4 days with 5% hematocrit (HCT) and 1% parasitemia without medium change every day with IgGs to final concentrations of 5, 10, 30, and 60/100 μl for anti-rBdp0 IgGs and 2.5 mg/ml for control anti-GST IgG. These different concentrations were prepared serially from a stock solution containing medium and purified IgGs. On the fourth day of cultivation, the inhibition growth percentage was calculated according to Rizk et al. (23). Two distinct trials were used to conduct the studies.

### *In vitro* Invasion Inhibition Assay

With slight adjustments, the *in vitro* invasion inhibition assay was carried out as previously described (26). *B. divergens* merozoites were collected from *B. divergens*-iRBCs at maximum parasitemia in 4-mm cuvettes using a Bio-Rad Gene Pulser with pulse controller with five intermittent high-voltage pulses (1.25 kV, 300, and 25F) with 10 s in ice between pulses using a Bio-Rad Gene Pulser with pulse controller (Bio-Rad, Laboratories, USA). In an RPMI 1640 medium, the recovered merozoites were combined with 2 mg/ml anti-rBdp0 IgG or anti-GST IgG. Cultures without antibodies were prepared as controls. Following that, 100 μl of the mixture, containing ~1 × 10^6^ free merozoites, was put into 96-well plates (Nunc) and incubated for 6 h at 37°C in a humidified multi-gas water-jacketed incubator. After 3 and 6 h, about 3,000 RBCs in Giemsa-stained smears were counted to determine parasitemia (maximum 1% after 6 h). For each antiserum concentration, two separate experiments were conducted in duplicate.

### Statistical Analyses

To evaluate significant differences among the means of all variables, one-way ANOVA was employed with GraphPad Prism software (GraphPad Prism version 5.0 for Windows; GraphPad Software, Inc., San Diego, CA, USA). Statistical significance was defined as a *p*-value of <0.05.

## Results

### Successful Expression of Bdp0 and Its Antigenic Role

**Figure 1:**
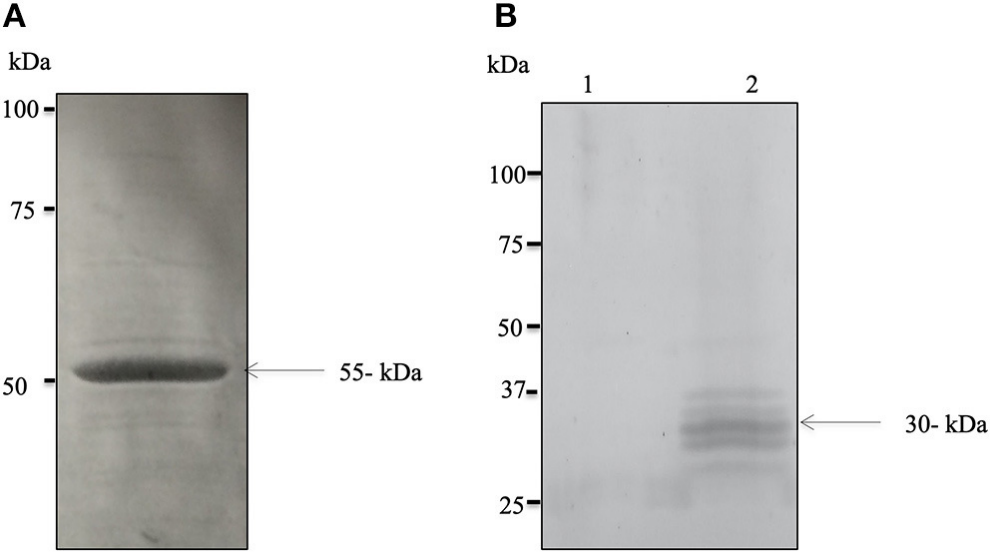
Expression and molecular characterization of the native BdP0. **(A)** SDS–polyacrylamide gel electrophoresis of purified recombinant rBdP0 in *E. coli*. SDS-PAGE recombinant protein stained with Coomassie Blue at a concentration of ~12% was utilized. The molecular weight of rBdP0 is 55 kDa. **(B)** Anti-rBdP0 serum produced in mice was used for Western blot of the lysates of non-infected (lane 1) and infected (lane 2) bovine erythrocytes. A distinct band at about 30 kDa was found in native BdP0.

The successful expression of cDNA for Bdp0 in *E. coli* was confirmed with sodium dodecyl sulfate–polyacrylamide gel electrophoresis (SDS-PAGE), and the rBdp0 had a molecular mass of 55 kDa (Figure 1A), including a 26-kDa GST tag. Polyclonal antibodies against –rBdp0 were produced in mice and were used to detect the native protein in the *B. divergens* lysate by Western blot analysis. In the *B. divergens* lysate, multiple bands with distinct bands corresponding to about 30 kDa native Bdp0 were found but not in non-infected bovine RBCs (Figure 1B, lanes 1 and 2). The appearance of multiple bands might be attributed to an excessive amount of lysate loaded onto the gel or antigen degradation due to proteolytic breakdown resulting in lower molecular weight products (29).

### Immune Reactivity of BdP0 and IFAT

**Figure 2:**
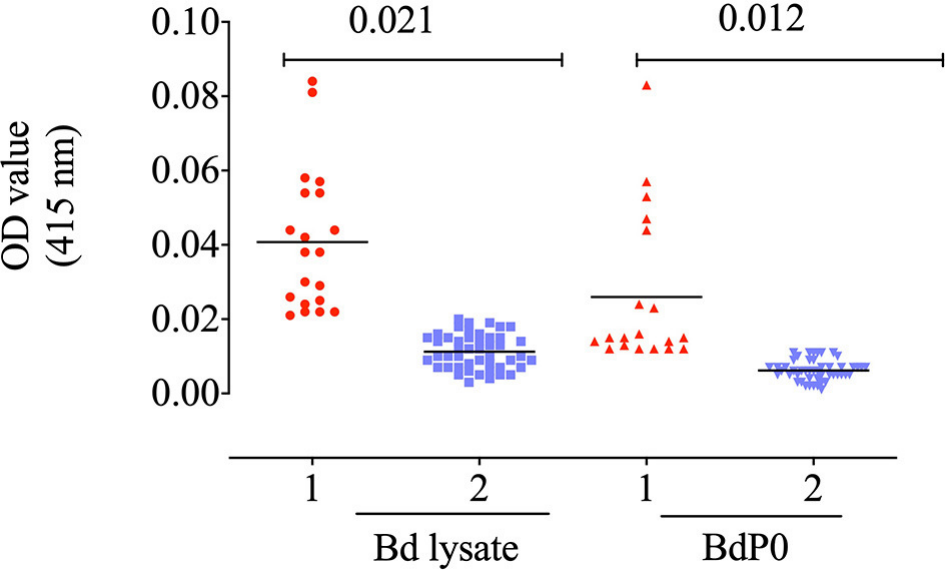
Immune reactivity of recombinant rBdP0. ELISA reactivity with bovine serum samples utilizing rBdEBP and *B. divergens* lysate. Positive sera are in lane 1; negative sera are in lane 2. A bar represents the cutoff value.

**Figure 3:**
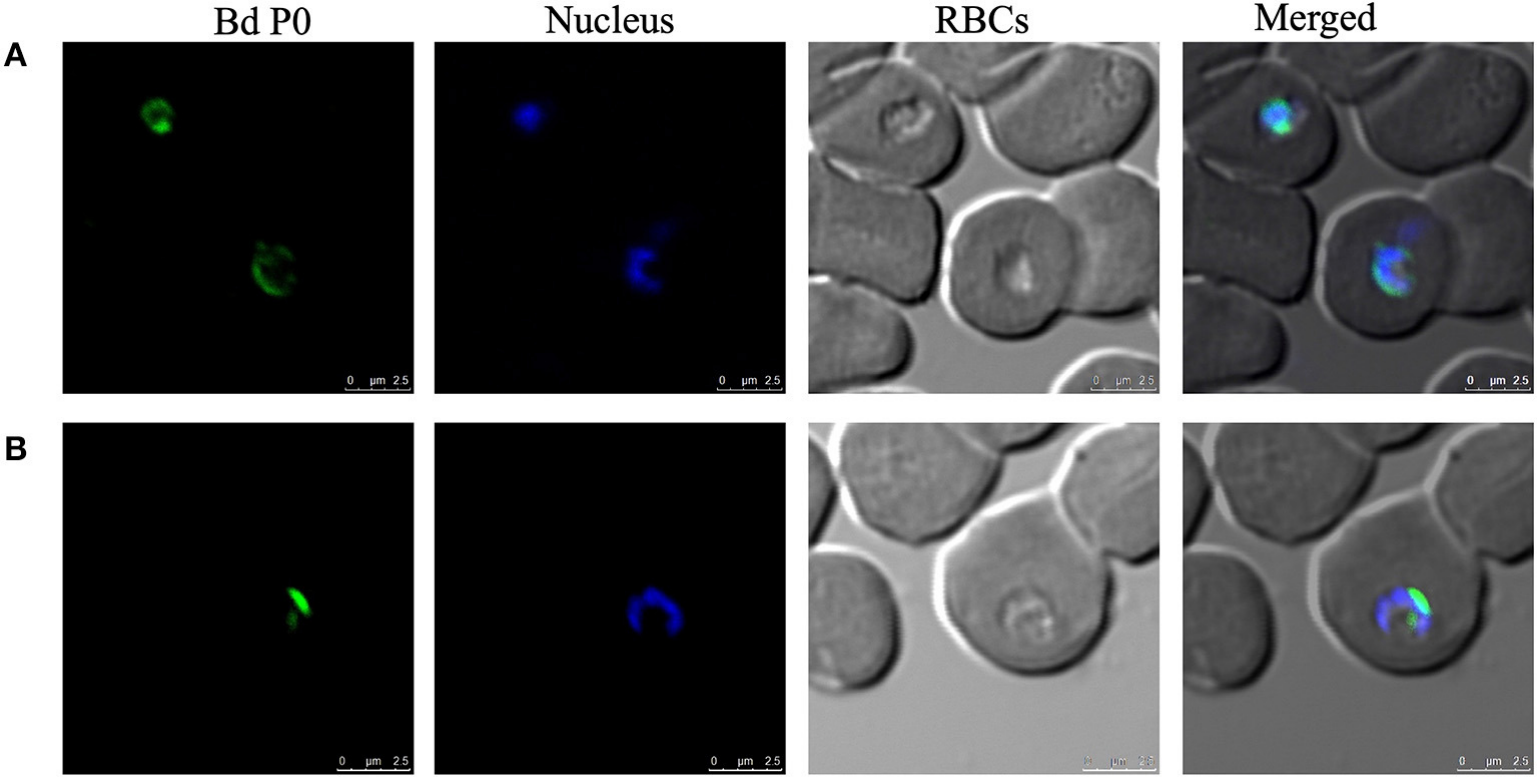
Cellular localization of BdP0 in the erythrocytic stage. Bdp0 in thin blood smears of *B. divergens*-parasitic RBCs stained with Bdp0-immune sera under confocal laser microscopy. Anti-rBdp0 serum reactivity with single intracellular parasite forms **(A)** and sequentially dividing forms **(B)**. Pre-immune and anti-GST sera were used as negative control sera to validate the test (data not shown). Scale bar: 7.5 μm.

The potential immunogenicity of BdP0 was evaluated with ELISA using a set of 68 bovine field samples, and a significant immune reactivity was obtained with 19 and 20 positive samples of rBdp0 and *B. divergens* lysate, respectively (Figure 2). Mice anti-rBdP0 serum were used to confirm the reactivity of Bdp0 with intracellular parasite single forms, which appear in the form of single round trophozoite (Figure 3A), and sequentially dividing forms appeared in the form of paired pyriforms, a stage formed by two attached pear-shaped sister cells (Figure 3B) by IFAT using confocal laser microscopy.

### Anti-rBdP0 Sera Inhibited the *in vitro* Growth and Invasion of *B. divergens*

**Figure 4:**
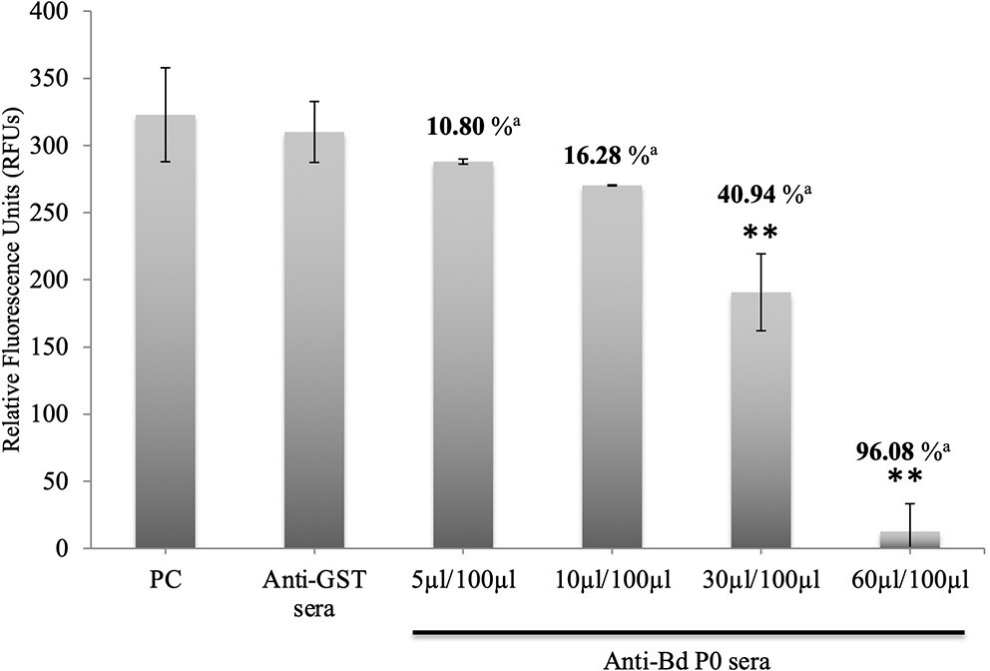
Growth inhibitory assay of *B. divergens* with different concentrations of IgGs. Fluorescence values were determined on the fourth day of culture. Statistically significant differences are indicated by asterisks (**p < 0.05) between the control group that received no antibody and groups that received different concentrations of Bd P0 antibodies sera. Results represent two repeated experiments. ^a^Inhibition percentage for each concentration.

**Figure 5:**
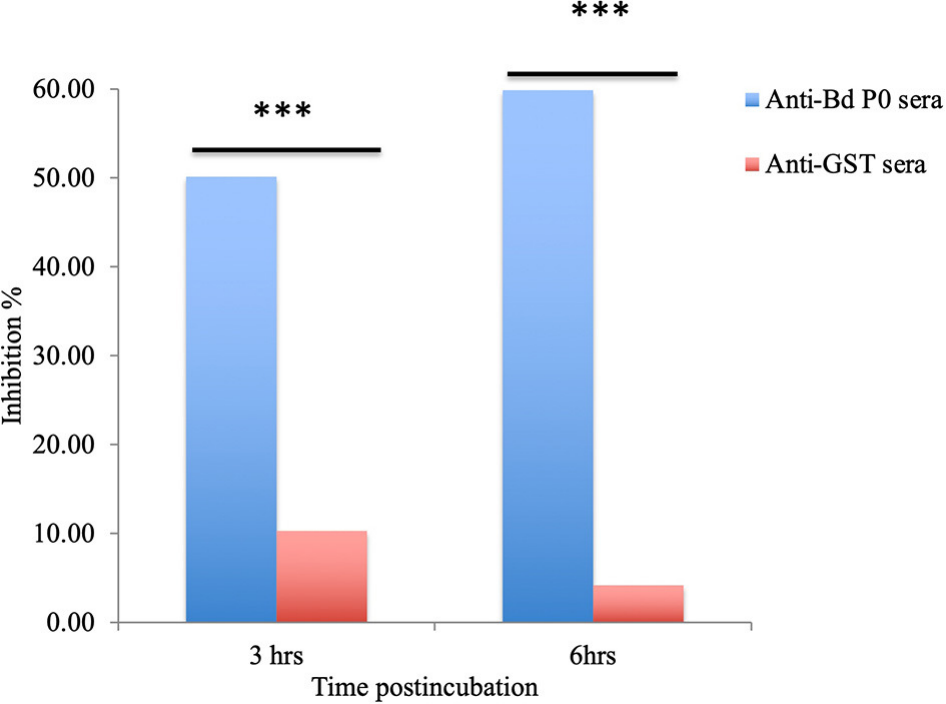
*Babesia divergens* invasion inhibition assay. After 3 and 6 h of culture, parasitemia was determined. ^***^p < 0.05 shows that the group that got BdP0 antibodies differed significantly from the group that received anti-GST IgG. The results are those of two separate investigations.

In a concentration-dependent manner, anti-rBdP0 sera suppressed parasite development. In contrast to the controls, emitted fluorescence signals were considerably inhibited (96.08%) at 60 μl/100 μl of mouse anti-rBdP0 for the course of the cultivation (Figure 4). In contrast, normal parasitemia growth was found in cultures using anti-GST immunoglobulins G (IgG) as a control antibody, which was similar to that seen in control cultures lacking antibodies. Anti-rBdP0 IgG was found to kill parasites and limit their *in vitro* growth, according to these findings. The question of whether rBdP0 is involved in RBC invasion is raised at this time. Free merozoites were pre-incubated with 2 mg/ml anti-rBdP0 IgG before being introduced to bovine RBCs to answer this question. Afterward, the parasitemia was determined 3 and 6 h post-culture. The parasitemia of invading parasites was significantly reduced (*p* < 0.05) in the anti-rBdP0-IgG-treated culture, with the highest inhibition (59.88%) occurring 6 h after culture (Figure 5). These findings revealed that anti-rBdP0 IgG's ability to neutralize free merozoites and disrupt their invasion was likely to blame for the growth inhibition in *B. divergens* culture.

## Discussion

The intraerythrocytic protozoan parasite *B. divergens* is the primary cause of bovine babesiosis in Europe. It can infect immunocompromised humans, causing medical problems such as fast fulmination and parasitemias of up to 70% (3). *B. divergens* is an intracellular parasite that must infect host RBCs in order to develop. Thus, invading the host cell to develop and multiply is the main crucial phase in the parasite's life cycle, and disease manifestations are linked to the parasite's proliferation inside infected erythrocytes (IEs). Recently, it was reported that the ribosomal protein including P0 shows an involvement in the cell invasion in addition to a regulatory role in DNA repair, cell development, and apoptosis (20). The multiple roles of the P0 protein in the ribosomes, nuclei, and cell surfaces presumably occur through interactions with other protein syntheses (30). For that, in this study, the antigenic characteristics of *B. divergens* ribosomal p0 proteins were discovered and described. The antiserum and recombinant protein were successfully generated. The antigenicity of rBdP0 was demonstrated by a robust reactivity to bovine field samples and *B. divergens* lysate, contrary to the previously identified *B. divergens* proteins, such as *B. divergens* apical membrane antigen-1 (BdAMA-1) and Rouen Bd1987 *B. divergens* Rhopty-associated protein 1 (RAP-1) (31), which have a limited immunological response to *B. divergens*-infected sera. BdP0 is functional at the merozoite stage and is involved in the invasion process, according to localization studies utilizing an immunofluorescence test with confocal laser microscopy. Our findings are consistent with prior findings indicating that P0 plays a key role in cell invasion in malaria parasites and *T. gondii* (19, 21, 32). *In vitro* culture, pure IgG from a polyclonal antibody produced against the *P. falciparum* PfP0N N-terminal domain completely suppressed parasite development (33). Furthermore, the passive transfer of anti-PfP0 into mice increased the mice's survival time when they were challenged with *P. yoelii* (32). *T. gondii* tachyzoites invading human cells were also inhibited by the same antibody (21). Additionally, passive-transfer immunization of anti-*B. gibsoni* IgG into SCID mice resulted in cross-protection against *B. microti* infection (20). Moreover, a *B. divergens* inhibition assay was used to confirm the inhibitory effect of specific antibody to rBdP0 on the *in vitro* growth and invasion of *B. divergens* parasites. In a concentration-dependent manner, significant reductions in parasite proliferation and invasion were reported. The anti-rBdP0 immune serum inhibits the parasitemia of invading parasites significantly after 6 h of incubation with the collected merozoites. Anti-rBdP0 IgG inhibits *B. divergens* invasion more effectively than anti-BdRAP-1 antibodies, which showed a 19% reduction in parasitemia in invading parasites after 36 h of incubation with free merozoites. The new translocation of P0 to the surface during the invasion phase is thought to be the mechanism behind Bdp0's inhibitory impact (17). This distinguishing feature is thought to be critical in the production of protective antibodies capable of neutralizing parasites (17). As a result, such an antigenic conserved protein has all of the protective properties to be a possible *B. divergens* vaccine. Anti-rBdEBP IgG likely neutralized free merozoites, causing them to lose their ability to infect host RBCs; this demonstrates that BdEBP binding to RBC receptors during the invasion stage is a common *Babesia* parasite strategy for infiltrating host cells.

Finally, the BdP0 protein, which was recently found, is active throughout cell division in the merozoite stage. Because of its surface location and the significant inhibitory effects of its specific antibody on *B. divergens* growth and invasion, BdP0 could be exploited as a molecular vaccine target for *B. divergens* infection. To assess the protective efficacy of this recombinant protein against infection in a laboratory model for *B. divergens* using the gerbil, more research is needed.

## Data Availability Statement

The raw data supporting the conclusions of this article will be made available by the authors, without undue reservation.

## Ethics Statement

The animal study was reviewed and approved by the Guiding Principles for the Care and Use of Research Animals Declared by Obihiro University of Agriculture and Veterinary Medicine were applied on all animal experiments used in this study. The protocol was approved by the Committee on the Ethics of Animal Experiments of Obihiro University of Agriculture and Veterinary Medicine (Permit number 23–26). The pathogen experiment's ID was 201708-4.

## Author Contributions

MR, SE-S, and II: conceptualization and visualization. SE-S and MR: data curation. MR, SE-S, and MT: formal analysis. MR, SS, MS-A, and II: funding acquisition. MR and II: investigation and validation. MR, SE-S, and MA: methodology. II: project administration and supervision. SE-S, MR, II, SS, MS-A, and HE: resources. MR, MT, and HE: software. MR, SE-S, and MS-A: writing—original draft. All authors have read and agreed to the published version of the manuscript.

## Funding

This study was supported financially by the Ministry of Education, Culture, Sports, Science, and Technology of Japan. MR was supported by a research grant fellowship for young scientists from the Japan Society for the Promotion of Science (JSPS) (ID no. P18091).

## Conflict of Interest

The authors declare that the research was conducted in the absence of any commercial or financial relationships that could be construed as a potential conflict of interest.

## Publisher's Note

All claims expressed in this article are solely those of the authors and do not necessarily represent those of their affiliated organizations, or those of the publisher, the editors and the reviewers. Any product that may be evaluated in this article, or claim that may be made by its manufacturer, is not guaranteed or endorsed by the publisher.
